# Hypothalamic-Pituitary Autoimmunity and Traumatic Brain Injury

**DOI:** 10.3390/jcm4051025

**Published:** 2015-05-19

**Authors:** Federica Guaraldi, Silvia Grottoli, Emanuela Arvat, Ezio Ghigo

**Affiliations:** 1 Division of Endocrinology, Diabetes and Metabolism, Department of Medical Sciences, University of Turin, Corso Dogliotti 14, Turin 10126, Italy; E-Mails: silvia.grottoli@unito.it (S.G.); ezio.ghigo@unito.it (E.G.); 2 Division of Oncological Endocrinology, Department of Medical Sciences, University of Turin, Corso Bramante 88, Turin 10126, Italy; E-Mail: emanuela.arvat@unito.it

**Keywords:** hypothalamic-pituitary autoimmunity, antipituitary antibodies, antihypothalamic antibodies, brain trauma injury

## Abstract

Background: Traumatic brain injury (TBI) is a leading cause of secondary hypopituitarism in children and adults, and is responsible for impaired quality of life, disabilities and compromised development. Alterations of pituitary function can occur at any time after the traumatic event, presenting in various ways and evolving during time, so they require appropriate screening for early detection and treatment. Although the exact pathophysiology is unknown, several mechanisms have been hypothesized, including hypothalamic-pituitary autoimmunity (HP-A). The aim of this study was to systematically review literature on the association between HP-A and TBI-induced hypopituitarism. Major pitfalls related to the HP-A investigation were also discussed. Methods: The PubMed database was searched with a string developed for this purpose, without temporal or language limits, for original articles assessing the association of HP-A and TBI-induced hypopituitarism. Results: Three articles from the same group met the inclusion criteria. Anti-pituitary and anti-hypothalamic antibodies were detected using indirect immunofluorescence in a significant number of patients with acute and chronic TBI. Elevated antibody titer was associated with an increased risk of persistent hypopituitarism, especially somatotroph and gonadotroph deficiency, while no correlations were found with clinical parameters. Conclusion: HPA seems to contribute to TBI-induced pituitary damage, although major methodological issues need to be overcome and larger studies are warranted to confirm these preliminary data.

## 1. Introduction

Traumatic brain injury (TBI) is a major and increasing cause of morbidity and mortality in adult and pediatric population in Western Countries and is associated with a significant financial and social burden [1]. 

According to the National Center for Injury Prevention and Control, in 2010 about 2.5 million emergency department visits (about 2.2. millions; 435,000 in children under 14 years of age; 70% rate increase from 2001 to 2010), hospitalizations (280,000) or deaths (more than 50,000 total; about 2700 in children) were caused by TBI in United States, with an estimated economic cost of nearly $76.5 billions [2]. 

Based on the Glasgow Coma Scale, the severity of TBI is categorized as mild (≥14), moderate (9–13) or severe (≤8) [3]. Severe and moderate traumas are often associated with long-term impairment of quality of life and disabilities in adults, and compromised development in children [1].

Falls are the leading cause of TBI (about 40% of the total, typical of children and older people), followed by blunt trauma (15%), road accidents (14%) and assaults/child abuse. Some recreational activities and sports—especially those involving contact/collision (*i.e*., boxing, football, soccer, ice hockey and the martial arts) or high speed (*i.e*., cycling, motor racing, equestrian sports, skiing and skating)—are often associated with mild and repetitive head trauma, secondary to concussion, with a cumulative effect on the development of pituitary dysfunction [4,5]. 

## 2. Traumatic Brain Injury and Pituitary Dysfunction

Hypopituitarism, firstly reported by Simmonds in 1914 and defined as the inability of the pituitary gland to provide sufficient hormones adapted to the needs of human body [6], has been increasingly reported in association with TBI, with the recent development of guidelines for appropriate screening and treatment [7,8,9]. At the same time, a large number of cases may remain unrecognized, possibly because of lack of physician awareness, underestimated associated health risks, and insidious, non-specific disease manifestations [8,9]. 

The reported prevalence of TBI-induced hypopituitarism ranges from 8% to 57% in children [1,10], and from 11% to 69% in adults [7,9]. Differences in prevalence and type of hormone disturbances could be secondary to the different patient demographics, study design, time of evaluation after TBI and protocol applied to the investigation of pituitary function [7,9]. 

Male patients in the third decade of life are typically affected [11]. A recent meta-analysis including more than 1000 adult patients, evaluated from 3 months to 7 years after trauma, reported a pooled prevalence of 27.5% of post-traumatic hypopituitarism [12]. Panhypopituitarism is always permanent, while partial hypopituitarism/isolated hormone deficiencies are in dynamic evolution and may occur at variable times, from a few days to decades after TBI, and may worsen or resolve during that time [7,8,9,11]. 

Most of the studies performed in children reported a high prevalence of isolated disorders, typically hyperprolactinemia and GH deficiency, occurring early and often resolved by 1 year after TBI [10,13]. 

Due to anatomical reasons, the anterior lobe is more frequently affected than the posterior lobe, and somatotroph and gonadotroph cells, located in the peripheral region, are at higher risk of damage. Indeed, blood supply to the posterior lobe is provided by short hypophyseal portal veins, while the anterior lobe is vascularized by the long hypophyseal portal vessels, passing through the diaphragma sellae, and the portal capillaries in the pituitary stalk, particularly vulnerable to mechanical compression and direct stalk injury [4,7]. In the case of pure anterior pituitary necrosis, a small area near the posterior lobe and the peripheral layer of anterior pituitary cells under the capsule, receiving blood supply from the capsule and not from portal vessels, survives. In the following years, the revascularization of this area by regenerated portal vessels stimulates cells growth, with repopulation of the anterior lobe and partial restoration of pituitary function [11]. 

Although the exact pathophysiology of TBI-induced hypopituitarism remains to be elucidated, it has been hypothesized that, in addition to the primary mechanical event, secondary insults (*i.e*., hypotension, hypoxia and increased intracranial pressure due to skull fractures, edema and hemorrhage) and changes in cerebral flow and metabolism can contribute to hypothalamic-pituitary damage [4]. 

More recently, persistent neuroinflammation and autoimmunity have been suggested as potential mechanisms involved in the development of TBI-induced hypopituitarism, especially in patients with genetic alterations [14]. Animal studies demonstrated a significant increase in the expression of interleukin IL-1b and glial fibrillary acidic protein in the cerebral cortex, hypothalamus and anterior pituitary following cortical contusion injury, which could be responsible for the activation and persistence of inflammatory processes, with subsequent neuronal diffuse damage and hypopituitarism [15]. Moreover, the synthesis of apolipoprotein E—a key protein for cell membrane repair particularly abundant in the hypothalamic-pituitary region—is upregulated within the central nervous system following injury, possibly to reduce the release of reactive oxygen species and inflammatory cytokines (*i.e*., TNF, IL-1 and IL-6) [16], with variable efficacy depending on the protein isoforms [17]. Finally, IgG antibodies against dying neurons in the injured brain [18] and serum autoreactive antibodies against neurons and basal lamina have been demonstrated in rats submitted to experimental TBI [19]. 

## 3. Hypothalamic-Pituitary Autoimmunity: Diagnostic Pitfalls

The role of autoimmunity in the development of hypopituitarism was first hypothesized in 1962 by Goudie and Pinkerton [20]. The authors reported a marked lymphocytic infiltration of the anterior pituitary and an enlarged thyroid and adrenal gland in a young woman who died of circulatory shock after surgery—probably secondary to adrenal insufficiency, as suggested by symptoms presented in the previous days—suggesting a generalized endocrine autoimmune disorder. Only seven additional autopsy-based cases of hypophysitis were reported up to 1978, reviewed by Bottazzo and Doniach who also hypothesized prolactin-secreting cells as the target of antipituitary antibodies (APA) [21]. Hypophysitis was first diagnosed by antemortem pituitary biopsy in 1980, and by MRI in 1988 [22]. In the following years, hypophysitis has been increasingly recognized based on clinical, biochemical and radiological features. At the same time, APA have been identified in association with various types of pituitary and extra-pituitary disorders [22,23], including extra-pituitary autoimmune disorders (*i.e*., thyroiditis [24,25], autoimmune polyendocrine syndrome [24,26,27], celiac disease in children [28], lupus erythematosus, multiple sclerosis and rheumatoid arthritis [29]), isolated anterior pituitary hormone deficiency [30], empty sella [31], Sheehan syndrome [32] and pituitary adenomas [33,34]. 

More recently, the presence of anti-hypothalamic antibodies (AHA) has been suggested in patients with isolated pituitary insufficiency of presumed idiopathic origin or in association with Sheehan’s syndrome [30,32,35].

At the same time, several important issues currently limit our understanding of pituitary autoimmunity and the usefulness of APA/AHA as a diagnostic and prognostic tool in the clinical field:

I. Lack of a reference method for APA investigation: Several methods have been applied to APA measurement, including indirect immunofluorescence (IIF), immunoblotting (IB), ELISA, fluid phase radioassays and antigen arrays. IIF reveals the presence of autoantibodies directed against an organ or tissue, and is useful when the autoantigen(s) recognized by autoantibodies are unknown. The main limitations are the low sensitivity and the subjective and weakly quantitative interpretation of results. IIF is one of the oldest but still most widely used technologies in the field of pituitary autoimmunity because of the relatively low costs, rapidity and ease of application, together with the uncertainty about antigen targets [23]. At the same time, interpretation of results obtained by IIF in the field of pituitary autoimmunity is significantly limited by (1) the high tissue background, secondary to natural tissue auto-fluorescence (from elastin and collagen contained in the blood vessels widely distributed in the pituitary gland, hemosiderin and lipofuscin, and non-specific antigen-antibody bindings), which could mask specific fluorescence signals, especially in case of weak signal intensity, but could also lead to false positive results; (2) the great methodological variability among studies, in terms of animal species of pituitary substrates, methods for tissue fixation, and inconsistent definitions of disease-associated staining patterns and antibody-titer thresholds to define positivity [22,23,34]. In that regard, a systematic revision of relevant articles reporting the presence of APA using IIF has been recently performed [36]: methodological limitations (*i.e*., suitability of pituitary tissue of various as substrate, blocking technique to reduce unwanted unspecific antigen-antibody binding, purification of secondary IgG-antibody, *etc*.) were first analyzed and an optimized protocol was then suggested with the aim of overcoming the identified issues. Some studies have been performed using ELISA or IB; the main limitations concerning interpretation of the results are the extremely low sample size, together with a high variability of disorders and targets investigated by the various authors, making results scarcely reproducible [22,37,38].

II. Pathogenic antigen target(s): Several candidates have been proposed (*i.e*., GH, alpha-enolase, pituitary gland specific factors 1a and 2 transcripts, secretogranin II, chorionic somatomammotropin and chromosome 14 open reading frame 166 and T-pit [22,23,34,37,38]), but no definite specific cellular targets of APA or AHA have been identified.

III. Role of APA on pathogenesis, clinical evolution and prognosis: The detection of APA and AHA, especially at high titers, has been suggested as a risk factor for the development of hypopituitarism in patients with autoimmune polyglandular syndromes [27,30,32,35]. On the contrary, the great majority of studies investigating association of APA and outcomes in various conditions found no clear associations [22,23,33,34,38], so the clinical significance of APA remain to be elucidated.

## 4. Hypothalamic-Pituitary Autoimmunity and Traumatic Brain Injury: Epidemiology and Clinical Implications

With the aim of reviewing literature assessing the association of pituitary autoimmunity and pituitary dysfunction in patients with TBI, we searched the PubMed database (http://www.ncbi.nlm.nih.gov/pubmed) using a string developed for that purpose—(traumatic brain injury) AND (autoimmunity OR (antipituitary antibodies)) AND (hypopituitarism OR (pituitary dysfunction))—with no language or time interval limits. Five articles were identified; only three of them, from the same group, were original and pertinent articles [39,40,41] and were included, while two were discarded (one was not pertinent, the other was a letter to the editor). A summary of study samples and findings is provided in Table 1. 

The involvement of APA in the pathogenesis of TBI-induced hypopituitarism was hypothesized by Tanriverdi and colleagues in 2008 [39]. Twenty-nine patients (25 M; age 36.5 ± 2.3 years), with no history of pituitary or autoimmune disorders or treatment with drugs affecting hypothalamic-pituitary function, were evaluated 3 years after acute TBI (62.1% mild, 20.7% moderate and 17.2% severe according to Glasgow Coma Scale) for basal and stimulated pituitary function (ACTH test 1 μg for ACTH deficiency; GHRH + GHRP6 and glucagon test for GH-deficiency). Patients and healthy, age 60 and sex-matched controls—recruited from volunteers who had no previous TBI or pituitary dysfunction—were also assessed for the presence of APA by indirect immunofluorescence, using baboon pituitary gland as substrate: serum dilution of 1:8 was defined as the cut-off for positivity; weak positivity was defined for titer ranging from 1:8 to 1:64, strong positivity for titer ranging from 1:64 to 1:256. APA were detected in 13 (44.8%) of the patients but in none of the controls. The risk of hypopituitarism was significantly higher in APA positive (6/13; 5 isolated GH-deficiency, 1 isolated ACTH-deficiency) than in APA negative (2/16) patients (*p* = 0.04; OR 2.25, 95% CI 1.1–4.6), especially for gonadal and somatotroph function (*p* < 0.05). Only patients strongly positive for APA showed pituitary dysfunction, with a significant positive correlation (r = 0.74; *p* = 0.004) between APA titer and GH response to GHRH+GHRP-6 test. At the same time no significant associations were found between clinical parameters and the presence of pituitary dysfunction and APA. 

These findings were confirmed by a subsequent study [41] performed with 25 patients (20 M; age 36.5 ± 2.3 years), from the original group of 29 evaluated at 36 months in the 2008 study [39], who were evaluated at 12 and 60 months (a 36 month-evaluation was available for 17 patients) after TBI (64% mild, 20% moderate and 16% severe based on Glasgow Coma Scale) for basal and stimulated pituitary function (using the same methods described above), and the presence of APA and AHA (by indirect immunofluorescence using baboon pituitary and hypothalamus, respectively). At a 12-month follow-up, 11 patients presented with GH-, 1 with TSH, 2 with FSH/LH and 4 with ACTH deficiency. Of the 17 patients evaluated at a 3-year follow-up, 4 had GH deficiency, while nobody presented with ACTH, LH/FSH or TSH deficiency. At a 60-month follow-up, 7 patients presented with GH deficiency, 1 with FSH/LH deficiency and another one with ACTH deficiency, while nobody presented with TSH deficiency. Recovery of pituitary function at 5-year follow-up was observed in patients with mild-moderate trauma but not in those with severe TBI. At the various follow-ups, GH deficiency was the most common alteration. 

**Table 1:** Summary of the studies assessing the association between hypothalamic-pituitary autoimmunity and traumatic brain injury (TBI)-induced hypopituitarism.

Author (year)	Total sample (*N*)	Type of TBI and N	Age at evaluation (yr; mean ± SD)	M:F	Glasgow Coma Scale (score; *N*)	CT findings (*N* of pt)	Hormone deficiency (*N*; type)	Time at evaluation after TBI (m; mean ± SD)	Evaluations performed	APA + (*N*)	AHA + (*N*)
Tanriverdi *et al*. (2008)	29	Road accident (18); fall (11)	36.5 ± 2.3	25:4	13–15: 18 9–12: 6 ≤8: 5	sub/epidural hemorrhage: 12 cranial vault fracture: 5 normal CT: 6 severe brain swelling: 2 basal skull fracture: 1	8 6 APA + (5 GH-D; 1 ACTH-D) 2 APA- (1 GH-D 1 ACTH-D)	36	Basal; GHRH+ GHRP6; ACTH 1 μg; glucagon test	13 (7 WP; 6 SP)	N/A
Tanriverdi *et al*. (2010)	61	Elite boxers (44 actively competing; 17 retired)	26 ± N/A	61:0	N/A	N/A	11 9 GH-D 5 ACTH-D	N/A		14	13
Tanriverdi *et al*. (2013)	25	Road accident, fall and other reasons	36.8 ± 2.1	20:5	13–15: 16 9–12: 5 ≤8: 4	N/A	*60 m*: 1 FSH/LH-D; 1 ACTH-D; 7 GH-D	12 and 60 (all pt); 36 (17 pt)		*60* m: 12 (7 WP; 5 SP)	*60* m: 15 (8 WP; 7 SP)

*Legend to table*: ACTH-D = adrenocorticotropic hormone; AHA = anti-hypothalamic antibodies; APA = anti-pituitary antibodies; antiCT = Computed Tomography; F = females; GH-D = growth hormone deficiency; m = months; M = males; *N* = patient number; pt = patients; SP = strong positivity; TBI = traumatic brain injury; TSH = thyreotroph stimulating hormone; WP = weak positivity; yr = years.

At a 5-year follow-up, 15 patients (60%) presented with AHA (8 weakly positive, 7 strongly positive), 12 (48%) with APA (7 weakly positive and 5 strongly positive), being strongly positive patients at higher risk of developing pituitary dysfunction (OR 5.3, 95% CI 1.3‒21.7 for AHA; OR 8.5, 95% CI 1.2‒64.3 for APA), while no significant correlation was found between the severity of TBI and antibody titer. Although functional and antibody evaluation was performed only in a limited number of patients at a 3-year follow-up, a strong antibody positivity was found in those with persistent pituitary deficiency from the third to the fifth year, but in none of those who recovered; pituitary dysfunction at the fifth year was more severe in patients with strongly antibody positivity at the third year as compared with negative or weakly positive ones (*p* = 0.0001). 

Finally, the presence of hypothalamic-pituitary autoimmunity was also demonstrated in patients suffering from chronic mild head trauma. In a recent study [40] hypothalamic-pituitary function and autoimmunity were assessed in 61 boxers (44 actively competing, 17 retired; mean age 26 years, range 17‒53) using the previously described methods. AHA and APA were found in 13 (21.3%; 4 weakly positive and 9 strongly positive) and 14 (22.9%; 6 weakly positive, 8 strongly positive) boxers, respectively, but in none of the controls. Of the 61 boxers, 11 (3 were active and 8 were retired) presented with hypopituitarism, 2 with ACTH deficiency, 6 with GH deficiency and 3 with both; 6 were AHA positive and 3 APA positive. Mean APA and AHA titers were higher in boxers with hypopituitarism (*p* < 0.027 and <0.006, respectively), with pituitary dysfunction significantly associated with AHA (OR 7.37, 95% CI 1.8‒30.8; *p* = 0.003), but not with APA. In the whole group, no significant correlation was found between autoantibodies and hormonal parameters, while in the group of retired boxers, AHA titer was associated positively with retirement age and inversely with GH peak at GHRH-GHRP6 test.

All together these studies confirm the high prevalence of TBI-associated hypopituitarism, especially in severe trauma and typically affecting the somatotroph axis, which dynamically changes during time, suggesting the involvement of hypothalamic-pituitary autoimmunity in its pathogenesis. At the same time, it should be remarked that the variability of type and severity of pituitary dysfunction could be secondary not only to functional reasons (*i.e*., severity and type of trauma, variations in antibody titers and levels of inflammatory markers, time of evaluation), but also to the method (*i.e*., basal *vs*. stimulated, and type of assay) applied to its investigation [7,9,12]. Indeed, the presence of anti-hypothalamic (more than anti-pituitary) antibodies at high titers appears to be a risk factor for the onset and persistence of TBI-induced hypopituitarism, in the context of chronic neuroinflammation. 

At the same time, the studies suffer from important limitations, mainly (1) small and heterogeneous patient samples; (2) cross-sectional (2 out of 3) studies; (3) unique experience from a single group; (4) absence of an early (*i.e*., first days after TBI) assessment of functional and immunological function to be used as baseline reference, and ongoing short-time periodical investigations to date their onset and follow their evolution; and (5) application of a non-optimized immunofluorescence method for the detection of anti-hypothalamic and anti-pituitary antibodies. 

Long-term, case control, prospective studies in larger cohorts, performed with an optimized immunofluorescence method are warranted to validate these preliminary data and, possibly, define the etiopathogenic and/or risk factors underlying antibody development after TBI and their clinical significance. 

## 5. Conclusions

In conclusion, anti-pituitary and anti-hypothalamic antibodies have been demonstrated in a significant number of subjects exposed to head trauma, suggesting the involvement of autoimmunity in the development and persistence of TBI-induced hypopituitarism, especially of isolated GH and ACTH deficiency. Although the autoimmune hypothesis is fascinating, etiopathogenic mechanisms underlying its development remain to be clarified and currently available experimental evidence is too scarce to routinely recommend specific screening in clinical settings. 
